# Complete Genome Sequences of Three Clinical Listeria monocytogenes Sequence Type 8 Strains from Recent German Listeriosis Outbreaks

**DOI:** 10.1128/MRA.00303-21

**Published:** 2021-05-06

**Authors:** Martin A. Fischer, Andrea Thürmer, Antje Flieger, Sven Halbedel

**Affiliations:** a FG11 Division of Enteropathogenic Bacteria and *Legionella*, German Consultant Laboratory for *L. monocytogenes*, Robert Koch Institute, Wernigerode, Germany; b MF2 Genome Sequencing, Robert Koch Institute, Berlin, Germany; University of Rochester School of Medicine and Dentistry

## Abstract

We report here the closed genome sequences of three clinical Listeria monocytogenes strains of multilocus sequence typing (MLST) sequence type 8 (ST8). These strains are representatives of three separate listeriosis outbreak clusters (Alpha1, Pi4, and Sigma1) that affected Germany between 2012 and 2020.

## ANNOUNCEMENT

The bacterium Listeria monocytogenes frequently causes foodborne outbreaks of invasive listeriosis (1). In Germany, diagnosing labs routinely send clinical isolates from human infections to the German Consultant Laboratory for L. monocytogenes for subtyping (2). Systematic genome sequencing of the isolates from patients and comparison with the isolates from possible food sources are then used for outbreak detection (2).

Three outbreaks recently detected were caused by PCR serogroup IIa clones belonging to multilocus sequence typing (MLST) sequence type 8 (ST8) (3, 4). Using 1,701-locus core-genome MLST (cgMLST), we further assigned cgMLST complex types 1248 (CT1248) (from the outbreak referred to as Alpha1), CT2521 (Sigma1), and CT5004 (Pi4; Table 1) to these clones (3–5). This report announces the completion of closed genome sequences of one representative strain for each of these outbreaks (Table 1).

**TABLE 1:** Key characteristics of the genomes sequenced in this study

Characteristic	Data for strain:		
	12-05460	18-04415	19-05816
Outbreak	Alpha1	Sigma1	Pi4
Source type	Clinical isolate	Clinical isolate	Clinical isolate
Source of isolation	Cerebrospinal fluid	Cerebrospinal fluid	Synovial fluid
Yr of isolation	2012	2018	2019
NCBI accession no.	CP063381	CP064843	CP063240
ENA accession no.	SAMEA104485072	SAMEA6798783	SAMEA7376280
Sequencing method	PacBio	MinION	MinION
ENA accession no. for raw long-read data	ERX4581156	ERX4581159	ERX4581158
ENA accession no. for raw short-read data	ERX2313070	ERX4056512	ERX4889691
Genome size (bp)	2,986,724	2,951,919	2,951,169
GC content (%)	37.95	37.96	37.96
No. of protein coding genes	2,922	2,965	2,881
No. of rRNA operons	6	6	6
No. of tRNA genes	67	67	67
Pathogenicity island	LIPI-1	LIPI-1	LIPI-1
Plasmid (GenBank accession no.)	None	pLMN1546 (CP064844)	None
PCR serogroup	IIa	IIa	IIa
ST^*a*^	ST8	ST8	ST8
CT^*b*^	CT1248	CT2521	CT5004
Outbreak duration	2012–2016	2014–2019	2017–2020
Reference(s)	3, 14	4	Unpublished

a ST, sequence type according to seven-locus MLST (15).

b CT, complex type according to 1,701-locus cgMLST (5).

L. monocytogenes strains were cultivated for 16 h in brain heart infusion (BHI) broth at 37°C, and chromosomal DNA was isolated by phenol-chloroform extraction, if not stated otherwise (6). The DNA quality and concentration were determined using a NanoDrop spectrophotometer (Thermo Fisher, Waltham, MA, USA). The species identity was confirmed by multiplex PCR (7).

For long-read sequencing of strain 12-05460 (Alpha1), a SMRTbell template library was generated (Pacific Biosciences, Menlo Park, CA, USA) using the BluePippin size selection system. Sequencing was performed on a PacBio RS system using one single-molecule real-time (SMRT) cell in 240-min movie run mode and components from the DNA sequencing kit 4.0 v2 (GATC Biotech, Constance, Germany). In total, 150,292 raw reads were obtained. Quality filtering and adapter trimming were carried out using SMRT Portal v2.3.0 software (Pacific Biosciences) with default parameters. Following this, 3,773 high-quality long reads remained (*N*_50_, 21,338 bp). Strain 12-05460 had also been sequenced previously using 2 × 300-bp paired-end Illumina sequencing, generating 1,614,580 reads (3).

Long-read libraries for strains 18-04415 (Sigma1) and 19-05816 (Pi4) were prepared using the SQK-RKB004 kit and sequenced on a MinION instrument using a 1D flow cell (Oxford Nanopore Technologies, Oxford, UK), generating 39,630 and 39,557 raw reads, respectively. The reads were quality filtered using NanoFilt (8) with default parameters. This resulted in 28,540 (*N*_50_, 7,332 bp) and 27,229 (*N*_50_, 12,075 bp) long reads, respectively. Strain 18-04415 had been sequenced previously on a MiSeq instrument using single-direction 1 × 150-bp chemistry, yielding 3,190,919 reads (4). For short-read sequencing, strain 19-05816 was cultivated for 16 h in BHI broth at 37°C. The isolation of chromosomal DNA and library preparation were performed as described previously (4). Sequencing was carried out on a NextSeq sequencer, in 2 × 150-bp mode, generating 3,318,986 reads. The raw reads were trimmed using Trimmomatic v0.36 (9).

All three genome sequences were assembled in Unicycler v0.4.8 (10) using long- and short-read sequences in hybrid assembly mode with standard parameters. The assembly resulted in one circular contig for isolates 12-05460 and 19-05816, representing their chromosomes, and two circular contigs, representing a chromosome and a plasmid, for isolate 18-04415. The genome sequences were rotated to the replication origin, and correct ring closure was confirmed by remapping the raw reads. The assembly statistics were calculated in QUAST v5.0.0 (11). The closed genome sequence of 12-05460 had a sequencing depth of 123-fold, a length of 2,986,724 bp, and a GC content of 37.95%. The genome sequence of 19-05816 had a sequencing depth of 165-fold, a length of 2,951,169 bp, and a GC content of 37.96%. The coverage for 18-04415 was 140-fold, and the genome was 2,951,919 bp long, with a GC content of 37.96%. This strain also contained the 86.6-kb plasmid pLMN1546 previously found in a Swiss outbreak strain (12). All sequences were submitted to NCBI and annotated using the NCBI Prokaryotic Genome Annotation Pipeline (13).

### Data availability.

The raw sequencing data and complete genome sequences are available at NCBI and ENA. All accession numbers can be found in Table 1.
